# Molecular Diagnostics for Lassa Fever at Irrua Specialist Teaching Hospital, Nigeria: Lessons Learnt from Two Years of Laboratory Operation

**DOI:** 10.1371/journal.pntd.0001839

**Published:** 2012-09-27

**Authors:** Danny A. Asogun, Donatus I. Adomeh, Jacqueline Ehimuan, Ikponmwonsa Odia, Meike Hass, Martin Gabriel, Stephan Ölschläger, Beate Becker-Ziaja, Onikepe Folarin, Eric Phelan, Philomena E. Ehiane, Veritas E. Ifeh, Eghosasere A. Uyigue, Yemisi T. Oladapo, Ekene B. Muoebonam, Osagie Osunde, Andrew Dongo, Peter O. Okokhere, Sylvanus A. Okogbenin, Mojeed Momoh, Sylvester O. Alikah, Odigie C. Akhuemokhan, Peter Imomeh, Maxy A. C. Odike, Stephen Gire, Kristian Andersen, Pardis C. Sabeti, Christian T. Happi, George O. Akpede, Stephan Günther

**Affiliations:** 1 Institute of Lassa Fever Research and Control, Irrua Specialist Teaching Hospital, Edo State, Nigeria; 2 Department of Virology, Bernhard-Nocht-Institute for Tropical Medicine, Hamburg, Germany; 3 Malaria Research Laboratories, College of Medicine, University of Ibadan, Ibadan, Nigeria; 4 Broad Institute, Cambridge, Massachusetts, United States of America; 5 Organismic and Evolutionary Biology, Harvard University, Cambridge, Massachusetts, United States of America; 6 Accident and Emergency Unit, Irrua Specialist Teaching Hospital, Edo State, Nigeria; 7 Department of Medicine, Irrua Specialist Teaching Hospital, Edo State, Nigeria; 8 Department of Obstetrics and Gynecology, Irrua Specialist Teaching Hospital, Edo State, Nigeria; 9 Department of Pediatrics, Irrua Specialist Teaching Hospital, Edo State, Nigeria; 10 Department of Family Medicine, Irrua Specialist Teaching Hospital, Edo State, Nigeria; 11 Department of Clinical Pathology, Irrua Specialist Teaching Hospital, Edo State, Nigeria; Tulane School of Public Health and Tropical Medicine, United States of America

## Abstract

**Background:**

Lassa fever is a viral hemorrhagic fever endemic in West Africa. However, none of the hospitals in the endemic areas of Nigeria has the capacity to perform Lassa virus diagnostics. Case identification and management solely relies on non-specific clinical criteria. The Irrua Specialist Teaching Hospital (ISTH) in the central senatorial district of Edo State struggled with this challenge for many years.

**Methodology/Principal Findings:**

A laboratory for molecular diagnosis of Lassa fever, complying with basic standards of diagnostic PCR facilities, was established at ISTH in 2008. During 2009 through 2010, samples of 1,650 suspected cases were processed, of which 198 (12%) tested positive by Lassa virus RT-PCR. No remarkable demographic differences were observed between PCR-positive and negative patients. The case fatality rate for Lassa fever was 31%. Nearly two thirds of confirmed cases attended the emergency departments of ISTH. The time window for therapeutic intervention was extremely short, as 50% of the fatal cases died within 2 days of hospitalization—often before ribavirin treatment could be commenced. Fatal Lassa fever cases were older (p = 0.005), had lower body temperature (p<0.0001), and had higher creatinine (p<0.0001) and blood urea levels (p<0.0001) than survivors. Lassa fever incidence in the hospital followed a seasonal pattern with a peak between November and March. Lassa virus sequences obtained from the patients originating from Edo State formed—within lineage II—a separate clade that could be further subdivided into three clusters.

**Conclusions/Significance:**

Lassa fever case management was improved at a tertiary health institution in Nigeria through establishment of a laboratory for routine diagnostics of Lassa virus. Data collected in two years of operation demonstrate that Lassa fever is a serious public health problem in Edo State and reveal new insights into the disease in hospitalized patients.

## Author Summary

In the past, diagnostic testing for Lassa fever patients in Nigeria has been performed nearly exclusively outside of the country. Patients thus were managed on-site based on clinical suspicion alone, posing risks to patients and health care workers and exhausting resources. To tackle this problem, we established a diagnostic PCR laboratory directly at a referral hospital serving a Lassa fever endemic area in Nigeria. Long-term collaboration between partners in the North and the South was crucial to implement this project. Training of laboratory staff in the partner institutions and on-site, mobilization of local human and financial resources, good management of the laboratory, a basic quality management and control system, and a stable supply chain for consumables and reagents were among the key factors for success. The laboratory reliably delivered results in a short turnaround time, despite some problems due to PCR contamination. The service has improved patient and contact management including treatment with ribavirin and led to better protection of health care workers against hospital-acquired infections. The data provide new insights into disease progression and a basis for further optimization of case management including supportive treatment.

## Introduction

Lassa fever is a viral hemorrhagic fever that was first described in 1969 in the town of Lassa in the North-East of Nigeria [1]. It is endemic in the West African countries of Sierra Leone, Guinea, Liberia, and Nigeria ([2], [3] and references therein). Cases imported to Europe indicate that Lassa fever also occurs in Côte d'Ivoire and Mali [4], [5]. The causative agent is Lassa virus, an RNA virus of the family *Arenaviridae*. Its natural host is the rodent *Mastomys natalensis* [6], [7], which lives in close contact to humans. *Mastomys* shed the virus in urine [8] and contamination of human food is a likely mode of transmission. The virus may be further transmitted from human to human, giving rise to mainly nosocomial epidemics with case fatality rates (CFR) of up to 65% [9]–[12]. However, most of the Lassa virus infections in the communities are probably mild [13].

Clinically, Lassa fever is extremely difficult to distinguish from other febrile illnesses seen in West African hospitals, at least in the initial phase [14], [15]. Gastrointestinal symptoms, pharyngitis, and cough are frequent signs. Late complications include pleural and pericardial effusions, facial edema, bleeding, convulsion, and coma. In the terminal stage patients often go into shock, although bleeding itself is usually not of a magnitude to produce shock [10], [14]–[22]. The only drug with a proven therapeutic effect in humans is the nucleoside analogue ribavirin. Drug efficacy decreases if treatment is commenced at day 7 or later [23], making early diagnostics critical for survival.

Lassa virus can be detected in blood at an early stage of illness. Death occurs about two weeks after onset of illness with fatal cases showing higher levels of viremia than those who survive. In survivors, virus is cleared from circulation about three weeks after onset of symptoms [24]–[26]. IgM and IgG antibodies are detectable only in a fraction of patients during the first days of illness, and patients with fatal Lassa fever may not develop antibodies at all [24], [26], [27]. Therefore, RT-PCR is a valuable tool for rapid and early diagnosis of Lassa fever [24], [25], [28], [29].

So far, diagnostic testing of samples from Lassa fever patients has been performed almost exclusively outside of Africa. Only the laboratory at the hospital in Kenema, Sierra Leone, which has become operational since 2004 (after civil war forced its closure in 1993), is able to perform Lassa fever testing for patients [30]. In Nigeria, the situation improved with the implementation of Lassa virus PCR testing at a research laboratory of the University of Lagos, which facilitated retrospective laboratory confirmation of Lassa fever cases in various parts of the country [31], [32]. However, none of the hospitals in the endemic areas of Nigeria has the capacity to perform Lassa virus tests. Case management is thus mainly based on non-specific clinical criteria [14], [15] and in the worst cases, health care workers became infected while they treated patients without knowing they had Lassa fever [32].

The Irrua Specialist Teaching Hospital (ISTH) has faced these challenges for many years. ISTH serves as a referral hospital in Edo State, one of the many Nigerian States with evidence of Lassa fever [1], [9], [12], [17], [32]–[37]. In 2001, ISTH was designated as a Centre of Excellence in the management of Lassa fever, along with two other federal tertiary health institutions. It set up awareness campaigns to sensitize hospital staff and the public to the severity of Lassa fever infection and need for treatment and prevention. Ribavirin was periodically supplied to the hospital by the Federal Ministry of Health and given to suspected cases. Prevalence and case fatality figures based on clinical suspicion and pilot laboratory investigations in 2003 and 2004 suggested a high incidence of Lassa fever in Edo State [31], but the true magnitude of the problem remained obscure. In 2007, the management of ISTH was dissatisfied with the level of response and attention given to Lassa fever and took bold steps to address the situation. Amongst these was the establishment of the Institute of Lassa Fever Research and Control (ILFRC). The rationale for the institute was based on the need to build capacity to adequately respond to the epidemics observed in the region in terms of manpower development and training, laboratory diagnosis, and adequate case management as well as the dire need for focused research and advocacy. A collaborative effort was made to establish a laboratory for molecular diagnostics of Lassa fever, which was considered crucial for appropriate case and contact management, including early treatment and postexposure prophylaxis with ribavirin [23], [38], [39]. The diagnostic and research laboratory was built in 2008 and started operation in September 2008. We describe here the establishment of a diagnostic service for Lassa fever and analyze the data recorded during two years of operation.

## Materials and Methods

### Ethics statement

The study was classified as a service evaluation and granted exemption from ethical review by the Research and Ethics Committee of ISTH. Lassa fever PCR diagnostics, patient management, and public health measures are part of routine clinical practice at ISTH. The choice of treatment or diagnostics was that of the clinician and patient according to professional standards or patient preference. Neither PCR diagnostics nor treatment with ribavirin was experimental in nature. All data described in the manuscript stem from existing records that have been generated as part of the regular clinical practice. The service was evaluated 2 years after implementation. Service evaluation is exempt from ethical review according to the National Code of Health Research Ethics, National Health Research Ethics Committee, Federal Ministry of Health, Nigeria.

### Case definition and ribavirin treatment

The following case definition, taking into account published signs and symptoms of Lassa fever, was used as a guideline for identifying suspected cases and requesting molecular testing for Lassa virus (from the diagnostic laboratory request form):

1. Patients with:
  - 38°C fever for at least 2 days
  - excluded typhoid fever and malaria negative or just 1+ in thick smear
  - and some or one of the following symptoms: chest pain, sore throat, headache, muscle pain, vomiting, and diarrhea.
2. Or: patients with fever who show bleeding or facial edema.
3. Or: patients with fever who do not respond to anti-malarials or antibiotics after 2 days of treatment.
4. Or: patients with fever who had contact with a confirmed Lassa fever case within the last three weeks.

However, as the laboratory provides routine diagnostic service for patients, there was flexibility in applying this case definition. Clinical experience and suspicion were taken into account as well. In addition, in the interest of time, Lassa virus testing was often performed before typhoid fever or malaria had been excluded. Samples were also sent from other parts of Nigeria for Lassa virus RT-PCR testing.

Ribavirin treatment was usually commenced on clinical grounds before laboratory testing. If RT-PCR was negative, it was terminated. However, in cases with a strong clinical suspicion for Lassa fever, ribavirin treatment was continued even if the RT-PCR was negative. If the RT-PCR was positive, treatment was continued or commenced.

### Diagnostic procedure

If a patient was suspected of having Lassa fever in any of the clinical or outpatient departments of ISTH, staff of ILFRC collected an EDTA blood sample. The blood was centrifuged and virus RNA was purified from plasma by using the diatomaceous earth method as described [40], [41]. In brief, 140 µl and 14 µl, respectively, of each plasma sample were mixed with 560 µl chaotropic lysis buffer AVL (Qiagen) containing 5.6 µg carrier RNA (Qiagen, no. 19073). AVL has been shown to inactivate enveloped RNA viruses [42]. The lysate was incubated at room temperature for 10 min. About 100 mg of diatomaceous earth (Sigma, no. D3877) and subsequently 560 µl of ethanol was added to the lysate and the slurry was incubated with vigorous agitation for 10 min at room temperature in a shaker. The diatomaceous earth was pelleted by centrifugation, and the pellet was washed three times, first with 500 µl of buffer AW1 (Qiagen, no. 19081), second with 500 µl of buffer AW2 (Qiagen, no. 19072), and finally with 400 µl of acetone (each washing step included vortexing with wash fluid, centrifugation for 2 minutes at maximum speed in a table top centrifuge, and removal of supernatant). The pellet was dried at 56°C for 20 minutes until the acetone was completely evaporated. To elute the RNA from the diatomaceous earth, the pellet was resuspended in 100 µl of water (Aqua ad injectabilia), the slurry was vortexed, incubated 1 minute at room temperature, centrifuged at maximum speed, and the supernatant was transferred to a new tube. The RNA was immediately used for PCR. The Lassa virus RT-PCR targeting the GPC gene was performed using QIAGEN OneStep RT-PCR Kit reagents (Qiagen, no. 210210 or 210212) as described [28]. The 25-µl assay contained 5 µl RNA, 0.6 µM primer 36E2 (ACC GGG GAT CCT AGG CAT TT), 0.6 µM primer LVS-339-rev (GTT CTT TGT GCA GGA MAG GGG CAT KGT CAT), 0.4 mM dNTP, 1× RT-PCR buffer, 1× Q-solution, and 1 µl enzyme mix. The reaction was performed in a Primus25advanced thermocycler (PeqLab, Erlangen, Germany) using the following temperature profile: 50°C for 30 min, 95°C for 15 min, followed by 45 cycles of 95°C for 30 s, 52°C for 30 s and 72°C for 30 s. All pre-PCR pipetting was performed with filter tips. PCR products were separated in a 1.5% agarose gel containing ethidium bromide and visualized by UV light. Gel images were recorded with a digital camera. As a positive control, inactivated culture supernatant of cells infected with Lassa virus strain CSF was used. All consumables, reagents, chemicals, kits, and plastic materials were purchased in Germany or the US and transferred to ISTH.

### Sequencing and phylogenetic analysis

PCR products generated in the diagnostics from September 2008 through February 2011 were stored at −20°C and sequenced retrospectively using primer 36E2. The automated base calling was proof-read by visual inspection of the electropherograms. A representative set of 35 sequences has been sent to GenBank and assigned the accession nos. JN651366-JN651400. Phylogenetic analysis included all novel GPC sequences (n = 204) as well as Lassa virus sequences available from GenBank by May 2011. The program jModelTest 0.1.1 [43] identified the general time-reversible model of sequence evolution with a gamma distribution of among-site nucleotide substitution rate variation (GTR+gamma) as the substitution model that best describes the data in the nucleotide sequence alignment of the partial GPC genes (284 taxa, 237 sites). The gamma+invariant sites model was not considered because it was not favored by jModelTest, and because the two parameters estimated under this model (the gamma distribution shape parameter and the proportion of invariant sites) are highly correlated and may be poorly estimated depending on the number of taxa [44]. Phylogenies were inferred by the Bayesian Markov Chain Monte Carlo method implemented in BEAST software [45] using the following parameters: GTR+gamma; 10^7^ steps with sampling every 10^5^th step; and two independent runs combined (effective sampling size >100 for all parameters). To avoid over-parameterization – considering that the sequences were short – simple molecular clock and demographic models were chosen, that is strict clock with mean substitution rate fixed at 1 and constant population size.

### Data management and statistical analysis

Demographic data as well as major symptoms at presentation were recorded on the request form that accompanied the blood sample. The department responsible for the patient provided clinical chemistry data generated in the Clinical Pathology Department of ISTH and data on treatment and outcome. Data were entered into a database (Excel, Microsoft) maintained at ILFRC. All patients from whom samples were processed in the Lassa fever diagnostics laboratory from January 2009 through December 2010 were included in the analysis. Each case was included only once; further testings on the same case were not considered. The data set was checked using plausibility criteria. A PCR result was corrected before analysis if the sequence of the PCR product indicated a false positive result, e.g. if the sequence corresponded to that of the positive control. Statistical comparison of unpaired groups was performed for continuous parameters with the Mann-Whitney test and for frequencies with two-tailed Fisher's Exact test. A critical p value of 0.01 was considered appropriate, given the large number of tests performed on the data set. The p value was further lowered to 0.001–0.0005 according to Bonferroni correction if multiple tests were conducted within one category (e.g. within the category profession).

## Results

### Establishment of a laboratory for molecular diagnosis of Lassa fever

**Figure 1:**
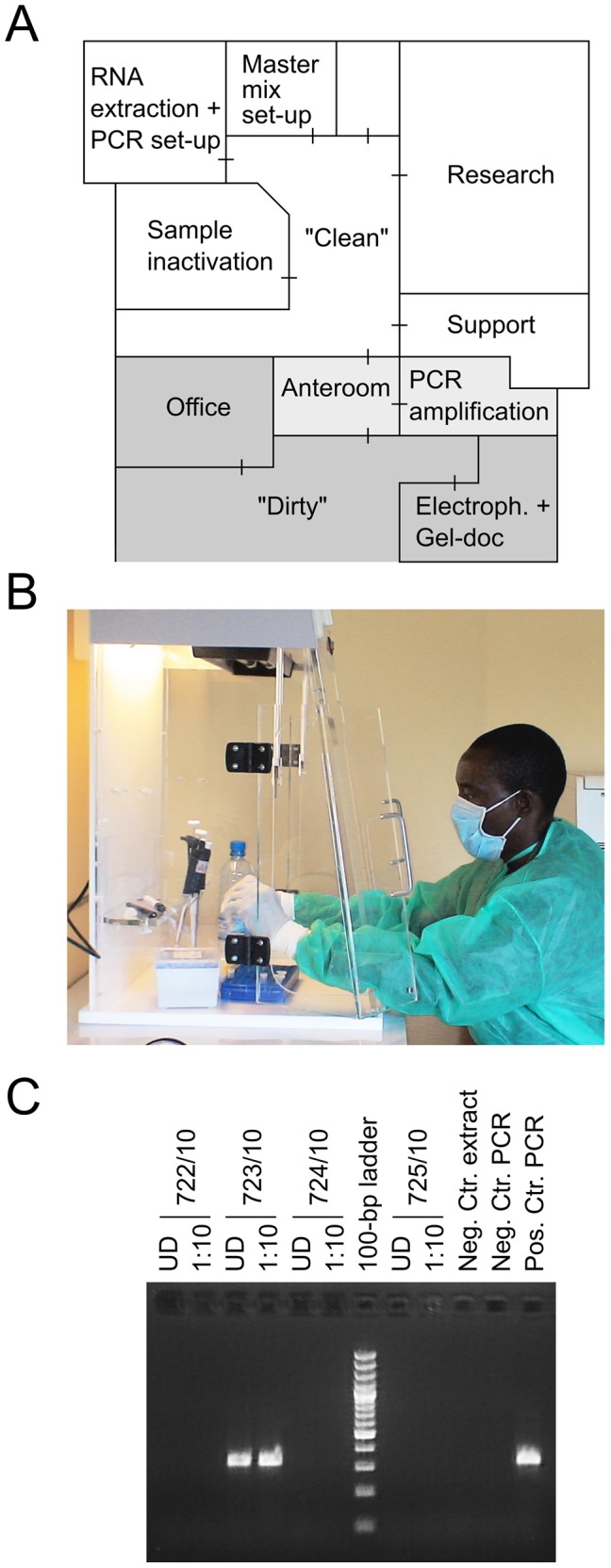
Molecular testing for Lassa virus at ISTH. (A) Outline of the diagnostic laboratory with pre- and post-PCR areas (“Clean” and “Dirty”, respectively). (B) Inactivation of plasma samples in a chaotropic buffer in a plexiglas box in the inactivation room. All sample manipulations were done behind a plexiglas shield. The box features a UV light source on top for decontamination. (C) Example of an RT-PCR result. From each patient sample, 140 µl and 14 µl were processed (lanes UD [undiluted] and 1∶10, respectively).

The laboratory was built in 2008 on the campus of ISTH. Equipment was provided by Bernhard-Nocht-Institute for Tropical Medicine (BNI), Harvard University, and University of Ibadan after initiating collaborations with the hospital in 2007. These partners also performed on-site trainings before and regularly during operation of the laboratory. In addition, staff of ILFRC was trained for 3 months in PCR technology at BNI in Hamburg and at Harvard University in Cambridge, Massachusetts. The laboratory started operation in September 2008. It is divided into three zones to minimize PCR contamination: a “clean” area for all pre-PCR manipulations, a “grey” area for amplification, and a “dirty” area for post-PCR manipulations (Figure 1A). The clean area features separate rooms for i) sample inactivation, ii) RNA extraction and PCR setup, and iii) mastermix preparation. The workflow starts with sampling blood using a closed syringe system. The sample is processed the same day or, if it arrives late, it is stored at 4°C and processed the next day. The syringe container is opened within a plexiglas box in the inactivation room and the plasma is mixed with chaotropic buffer to inactivate the virus and prepare RNA, or aliquoted for storage at −20°C (Figure 1B). From each plasma sample, 140 µl and 14 µl are inactivated and processed separately. The reason for testing two different volumes per sample is to avoid false negative results due to PCR inhibition, which appears to be a particular problem with samples from patients with severe hemorrhagic fever [46]. If the undiluted sample (140 µl) would be false negative due to inhibition, the 1/10-volume sample (14 µl) was expected to be positive due to the dilution of the inhibitor. In addition, running two PCRs in parallel on a patient sample enhances the reliability of the diagnostic process, as in most Lassa fever cases both samples were positive due to the high virus load (see below). RNA is extracted from samples and a negative control by the diatomaceous earth method [40] in the RNA extraction room. This method is inexpensive, and has been demonstrated to recover RNA over a broad concentration range with high efficacy [47]. The RT-PCR mastermix is prepared in the mastermix room and transferred to the extraction room for setting up the reaction. The closed PCR vials are transferred to the PCR thermocycler in the “grey” area. After completion of the PCR run, the closed vials are transferred to the detection room in the “dirty” area for agarose gel electrophoresis and gel documentation. PCR results are technically evaluated and transmitted to the clinics on a report form (Figure 1C). If either the undiluted or the 1/10-volume sample was positive, additional tests were performed to exclude PCR contamination. Gel pictures were transmitted to BNI staff via the internet for process monitoring. Standard laboratory procedures were defined in a set of quality management documents.

### Laboratory testing

During 2009 through 2010, the laboratory has processed blood samples of 1650 patients. Testing a second sample was requested for 57/1650 patients; all other patients were tested once. Retrospective verification of positive test results by sequencing the PCR products revealed that in 13/1650 cases (0.8%), the result was probably false positive result due to PCR contamination. The sequences of the corresponding PCR fragments matched exactly that of the positive control or the sequence of a highly positive sample processed before. For data analysis, these samples were re-classified as negative, as well as samples which have initially been reported as indeterminate (n = 12; e.g. faint PCR signals that were not confirmed in a second blood sample). Applying these criteria, 1452 cases (88%) were Lassa RT-PCR negative, and 198 cases (12%) were positive. Undiluted and 1/10-volume RNA extract were positive in 138 (70%) cases and one of both was positive in 60 (30%) of the Lassa fever cases. The outcome was known for 170 cases with confirmed Lassa fever: 61 died and 109 survived. Thus, the CFR is 31% if calculated based on all Lassa fever patients or 36% if calculated based on cases with known outcome.

### Demographic data of patients (Table 1)

**Table 1:** Demographic data for the patients.

			Lassa virus PCR-positive		
Category	All patients tested (n = 1650)	Lassa virus PCR-negative (n = 1452)	All (n = 198)	Survived (n = 109)^a^	Died (n = 61)^b^
Age (years)					
Median (Q25–Q75)	28 (18–43)	28 (17–42)*	32 (23–46)*	30 (22–40)#	40 (24–58)#
N data points	1579	1384	195	108	60
Gender, n (%)					
Female	809 (50)	713 (50)	96 (49)	57 (53)	27 (44)
Male	823 (50)	722 (50)	101 (51)	51 (47)	34 (56)
N data points	1632	1435	197	108	61
Profession, n (%)					
Child 0–4 years	170 (12)	156 (12)	14 (8.5)	8 (8.9)	5 (10)
Child 5–9 years	103 (7.3)	95 (7.6)	8 (4.8)	4 (4.4)	2 (4.0)
Child 10–15 years	72 (5.1)	65 (5.2)	7 (4.2)	3 (3.3)	3 (6.1)
Student	256 (18)	224 (18)	32 (19)	24 (26)	3 (6.1)
Trader	137 (9.7)	118 (9.5)	19 (11)	10 (11)	7 (14)
Civil servant	125 (8.8)	115 (9.2)	10 (6.1)	4 (4.4)	4 (8.1)
Healthcare worker	98 (6.9)	93 (7.4)	5 (3.0)	3 (3.3)	–
Farmer	98 (6.9)	80 (6.4)	18 (10)	8 (8.9)	9 (18)
Business	61 (4.3)	54 (4.3)	7 (4.2)	1 (1.1)	3 (6.1)
Workman/engineer	50 (3.5)	42 (3.3)	8 (4.8)	6 (6.7)	–
Teacher/lecturer	45 (3.2)	38 (3.0)	7 (4.2)	5 (5.6)	–
House wife	32 (2.2)	28 (2.2)	4 (2.4)	–	4 (8.1)
Pensioner	22 (1.5)	17 (1.3)	5 (3.0)	2 (2.2)	2 (4.0)
Tailor	20 (1.4)	19 (1.5)	1 (0.6)	1 (1.1)	–
Applicant	19 (1.3)	17 (1.3)	2 (1.2)	2 (2.2)	–
Driver	19 (1.3)	17 (1.3)	2 (1.2)	1 (1.1)	–
Office/academics	18 (1.2)	14 (1.1)	4 (2.4)	1 (1.1)	2 (4.0)
Banking	10 (0.7)	9 (0.7)	1 (0.6)	1 (1.1)	–
Clergyman	10 (0.7)	5 (0.4)	5 (3.0)	1 (1.1)	4 (8.1)
Hair dresser	9 (0.6)	7 (0.5)	2 (1.2)	2 (2.2)	–
Security/police	8 (0.5)	6 (0.4)	2 (1.2)	1 (1.1)	1 (2.0)
Other	24 (1.7)	23 (1.8)	1 (0.6)	1 (1.1)	–
N data points	1406	1242	164	89	49
State of Origin, n (%)					
Edo	1439 (89)	1268 (89)	171 (88)	94 (87)	52 (88)
Ondo	63 (3.9)	48 (3.4)	15 (7.7)	13 (12)	1 (1.6)
FCT	29 (1.8)	26 (1.8)	3 (1.5)	–	2 (3.3)
Ebonyi	21 (1.3)	20 (1.4)	1 (0.5)	–	1 (1.6)
Nasarawa	2 (0.1)	–	2 (1.0)	–	2 (3.3)
Other	49 (3.0)	48 (3.4)	1 (0.5)	–	1 (1.6)
N data points	1603	1410	193	107	59
LGA of Edo, n (%)					
Esan West	458 (31)	407 (32)	51 (29)	33 (35)	9 (17)
Esan Central	361 (25)	322 (25)	39 (22)	22 (23)	12 (23)
Esan North East	195 (13)	168 (13)	27 (15)	15 (15)	9 (17)
Etsako West	114 (7.9)	93 (7.3)	21 (12)	9 (9.5)	8 (15)
Owan West	59 (4.1)	49 (3.8)	10 (5.8)	5 (5.3)	5 (9.6)
Esan South East	52 (3.6)	47 (3.7)	5 (2.9)	2 (2.1)	1 (1.9)
Igueben	43 (2.9)	40 (3.1)	3 (1.7)	2 (2.1)	1 (1.9)
Akoko Edo	35 (2.4)	33 (2.6)	2 (1.1)	–	2 (3.8)
Owan East	27 (1.8)	24 (1.8)	3 (1.7)	1 (1.0)	2 (3.8)
Etsako Central	21 (1.4)	20 (1.5)	1 (0.5)	1 (1.0)	–
Uhunmwode	19 (1.3)	12 (0.9)	7 (4.0)	3 (3.1)	2 (3.8)
Etsako East	12 (0.8)	11 (0.8)	1 (0.5)	–	1 (1.9)
Egor	4 (0.2)	3 (0.2)	1 (0.5)	1 (1.0)	–
Other	39 (2.7)	39 (3.0)	–	–	–
N data points	1439	1268	171	94	52

Due to a variable number of missing values, the number (n) of data points that were included in the analysis is indicated with each category.

Abbreviations: FCT, Federal Capital Territory; LGA, Local Government Area; Q25, lower quartile, 25% of the data lie below this value; Q75, upper quartile, 75% of the data lie below this value.

a Patients who were discharged after recovery.

b Patients who died during hospitalization.

* p<0.01 (PCR-negative vs. PCR-positive).

# p<0.01 (PCR-positive survived vs. PCR-positive died).

**Figure 2:**
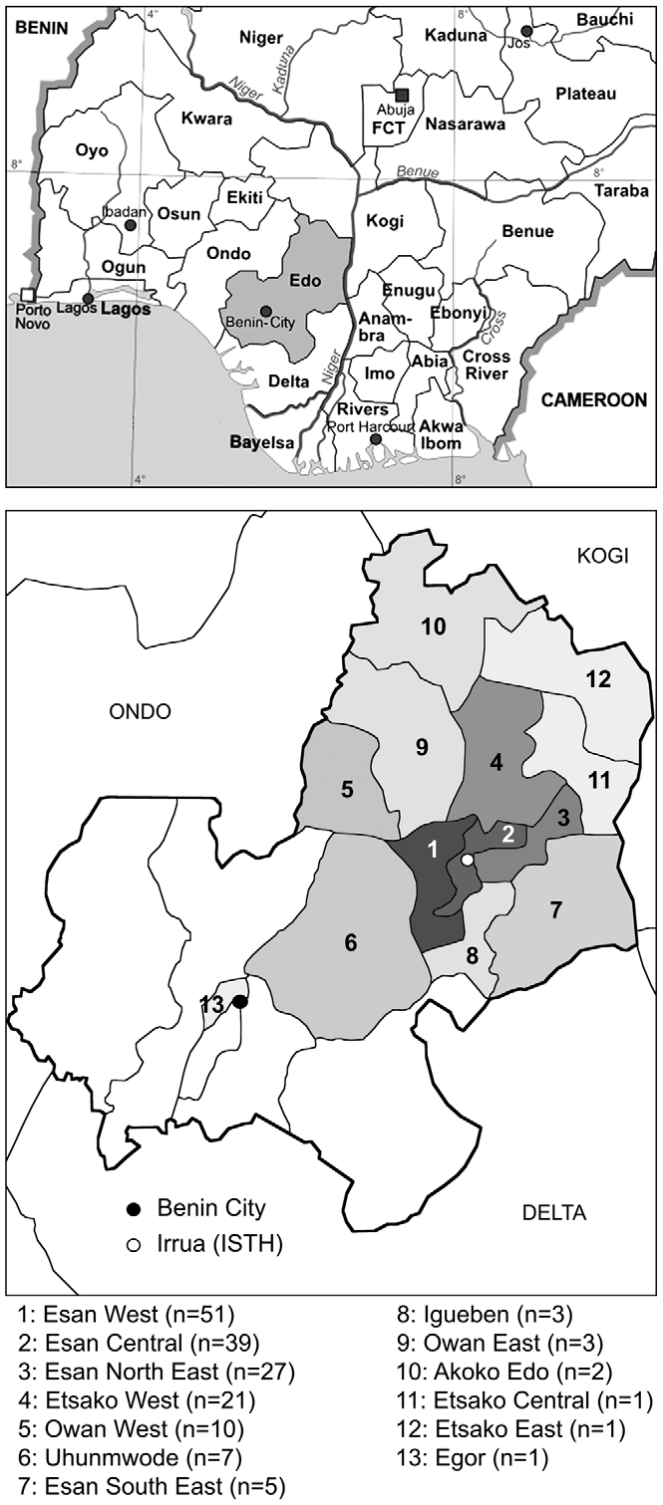
Geographic origin of Lassa fever cases. Top: Map of the southern part of Nigeria showing location of Edo State. Most of the samples came from Edo State and the neighboring Ondo State. FCT, Federal Capital Territory. Bottom: Map of Edo State with borders of the Local Governmental Areas (LGA). LGAs with confirmed Lassa fever cases are shaded from light to dark grey depending on the number of cases. The names of the LGAs with case number in parentheses are given below the map.

**Figure 3:**
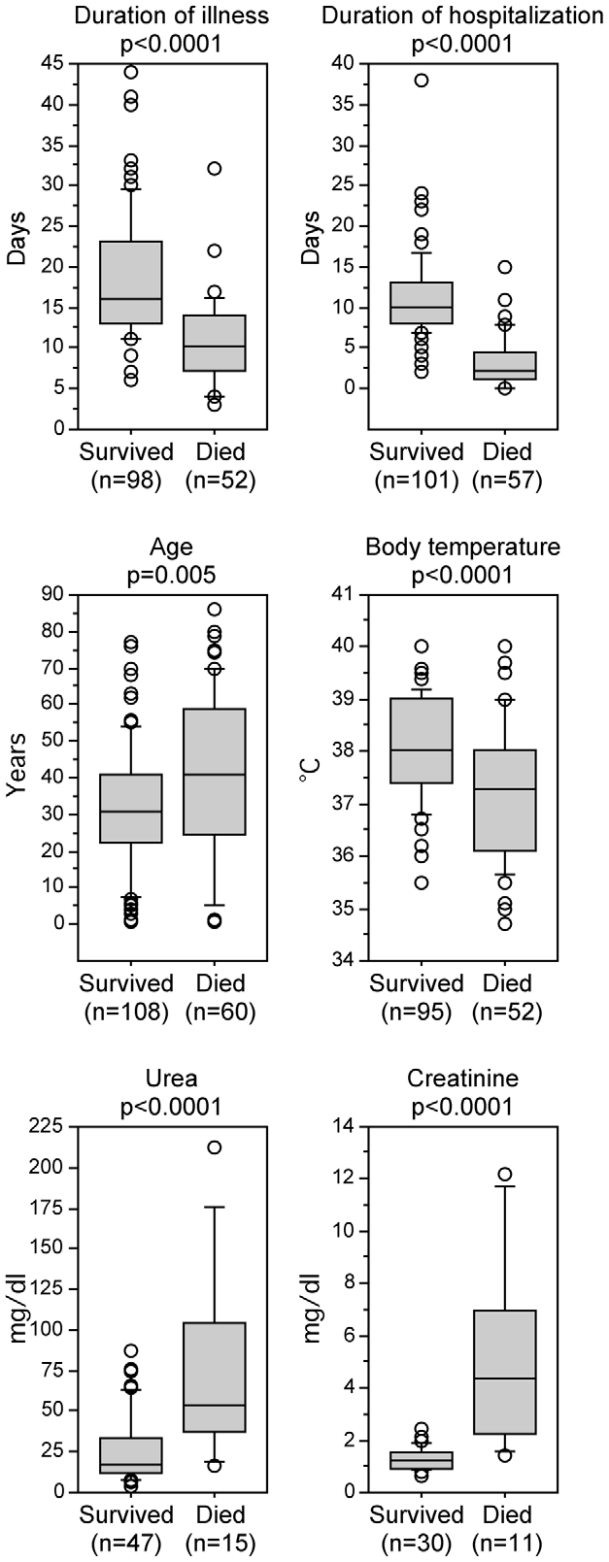
Box-plot representation of statistically significant differences between survivors and fatal cases of Lassa fever. Urea and creatinine blood levels were drawn at admission. Comparison of unpaired groups was performed with the Mann-Whitney test. The number of cases per group is given below the plots in parentheses.

The vast majority of the patients came from Edo State, in particular from the Local Governmental Areas (LGA) surrounding ISTH, namely Esan West, Esan Central, Esan North East, Etsako West, and Owan West (Figure 2). Several patients also came from the neighboring state of Ondo, and a few samples were sent from other parts of Nigeria. The median age of Lassa fever patients was 32 years. Those who died from the disease were older than those who survived (p = 0.005) (Figure 3). The proportion of males and females was equal, and no association with outcome was observed. Children and students made up about one third of the patients. Adult patients had various professions and no specific profession was associated with the outcome of Lassa fever. When comparing the demographic data of Lassa fever negative versus positive patients, no statistically significant differences were observed with the exception of age, which tended to be higher among the positive patients (p = 0.006).

### Hospital and disease-related data (Table 2)

**Table 2:** Hospital and disease-related data.

			Lassa virus PCR-positive		
Category	All patients tested (n = 1650)	Lassa virus PCR-negative (n = 1452)	All (n = 198)	Survived (n = 109)^a^	Died (n = 61)^b^
Time from onset to presentation (days)					
Median (Q25–Q75)	5 (2–11)	5 (2–11)*	6.5 (4–10)*	6 (3–10)	7 (4–10)
N data points	1273	1099	174	97	51
Time from presentation to sampling for PCR (days)					
Median (Q25–Q75)	0 (0–1)	0 (0–1)	0 (0–1)	0 (0–1)	0 (0–1)
N data points	1481	1292	189	103	59
Duration of hospitalization (days)^c^					
Median (Q25–Q75)	6 (3–10)	5 (3–10)*	8 (3–11)*	10 (8–13)##	2 (1–4)##
N data points	860	694	166	101	57
Duration of illness (days)^d^					
Median (Q25–Q75)	12 (7–18)	11 (7–18)*	15 (11–21)*	16 (13–23)##	10 (7–14)##
N data points	787	628	159	98	52
Department, n (%)					
Emergency Adults	518 (32)	420 (29)**	98 (50)**	54 (50)	32 (54)
Emergency Children	247 (15)	223 (15)	24 (12)	13 (12)	10 (16)
Medical	266 (16)	238 (16)	28 (14)	16 (14)	11 (18)
Outpatient	316 (19)	300 (21)**	16 (8.2)**	12 (11)	1 (1.7)
Gyn & Obstetrics	60 (3.7)	54 (3.8)	6 (3.1)	4 (3.7)	–
Pediatrics	34 (2.1)	30 (2.1)	4 (2.0)	1 (0.9)	–
Surgery	17 (1.0)	16 (1.1)	1 (0.5)	1 (0.9)	–
Ear Nose Throat	20 (1.2)	20 (1.4)	–	–	–
Staff/HCW	28 (1.7)	26 (1.8)	2 (1.0)	1 (0.9)	–
Intensive Care	1 (0.1)	1 (0.1)	–	–	–
Not ISTH	98 (6.1)	83 (5.8)	15 (7.7)	5 (4.6)	5 (8.4)
N data points	1605 (100)	1411 (100)	194 (100)	107 (100)	59 (100)
Axillary body temperature on presentation (°C)^e^					
Median (Q25–Q75)	37.5 (36.7–38.4)	37.5 (36.6–38.3)*	37.9 (37.0–38.8)*	38.0 (37.4–38.9)##	37.2 (36.1–38.0)##
N data points	1338	1164	174	95	52
Outcome, n (%)					
Death	185 (13)	124 (10)**	61 (32)**	–	61 (100)
Referred	26 (1.9)	21 (1.7)	5 (2.6)	–	–
DAMA	38 (2.7)	33 (2.8)	5 (2.6)	–	–
Not admitted	431 (31)	425 (36)**	6 (3.2)**	–	–
Discharged	685 (50)	576 (48)	109 (58)	109 (100)	–
N data points	1365	1179	186	109	61
Ribavirin, n (%)					
Yes	276 (19)	107 (8.5)**	169 (92)**	107 (100)##	41 (77)##
No	1163 (81)	1149 (91)	14 (7.6)	–	12 (23)
N data points	1439	1256	183	107	53

Due to a variable number of missing values, the number (n) of data points/patients that were included in the analysis is indicated with each category.

Abbreviations: DAMA, discharge against medical advice; Q25, lower quartile, 25% of the data lie below this value; Q75, upper quartile, 75% of the data lie below this value.

a Patients who were discharged after recovery.

b Patients who died during hospitalization.

c Time from presentation to either death in hospital, DAMA, referral to another hospital, or discharge after recovery.

d Time from onset of illness to either death in hospital, DAMA, referral to another hospital, or discharge after recovery.

e normal range 35.5–37.0°C [48].

* p<0.01 (PCR-negative vs. PCR-positive).

** p<0.0001 (PCR-negative vs. PCR-positive).

## p<0.0001 (PCR-positive survived vs. PCR-positive died).

Patients presented at the hospital 5 days (median) after onset of symptoms. Lassa fever positive patients presented slightly later than those who tested negative (median difference 1.5 days, p = 0.009). A blood sample for Lassa fever diagnostics was taken from 75% of the patients at the day of presentation or the following day without differences among the groups. Lassa fever patients stayed longer in hospital and had a longer duration of illness than those who tested negative (p = 0.002 and p = 0.01, respectively). Clear differences in these two categories were observed between fatal cases of Lassa fever and survivors. Survivors stayed 10 days in hospital and had duration of illness of 16 days, while patients died from Lassa fever 2 days after admission and 10 days after onset of symptoms (figures are median; p<0.0001 and p<0.0001, respectively) (Figure 3). The time window for therapeutic intervention was extremely short: 25% of the fatal cases died one day after admission and the same day the blood sample was taken for Lassa virus RT-PCR; 75% died within 4 days of hospitalization and within 3 days after sampling for PCR.

Patients with signs of Lassa fever were seen in virtually all clinical and outpatient departments of ISTH. The vast majority of requests for Lassa fever testing came from the emergency departments, the medical wards, and the outpatient departments. Lassa fever patients were significantly more frequent among patients attending the adult emergency department (detection rate 19%; p<0.0001) while they were underrepresented among patients attending the outpatient departments (detection rate 5%; p<0.0001). Thus, nearly two thirds of laboratory-confirmed Lassa fever cases were seen in the emergency departments of ISTH.

Median axilliary body temperature recorded at the time of presentation was 37.5°C for all patients tested. Thus, most of them had a body temperature lower than indicated in the case definition (≥38°C). Body temperature was slightly higher for those who tested positive (p = 0.003). However, patients who had a fatal outcome had 0.8°C lower temperature (median difference) than those who survived (p<0.0001) (Figure 3). Indeed, nearly half of the fatal cases had normal axilliary temperature (35.5°C–37°C) [48] and 75% had ≤38°C. A few had hypothermia (≤35.5°C).

The CFR among Lassa fever patients was three times higher than the fatality rate among patients who tested negative for Lassa fever (p<0.0001), and negative patients were 10-times less frequently admitted to hospital than positive patients (p<0.0001). Ribavirin treatment was given to nearly all laboratory-confirmed Lassa fever patients, although several patients who tested negative received the drug as well, at least initially. While all survivors received the drug, 23% of Lassa fever patients with fatal outcome did not receive ribavirin because they died the day of presentation or the next day.

### Virological and clinical data for Lassa fever patients (Table 3)

**Table 3:** Virological and clinical data for Lassa fever patients.

	Lassa virus PCR-positive	
Category	Survived (n = 109)^a^	Died (n = 61)^b^
Lassa virus load on presentation (score)^c^		
1+	29 (27)#	4 (6.5)#
2+	80 (73)	57 (93)
N data points	109	61
Symptoms and signs on presentation		
Fever	100 (94)	46 (88)
Vomiting	54 (50)	18 (34)
Headache	52 (49)	20 (38)
Abdominal pain	42 (39)	16 (30)
Sore throat	18 (17)	6 (11)
Cough	16 (15)	13 (25)
Chest pain	15 (14)	5 (9.6)
Joint/body pain	14 (13)	8 (15)
Diarrhea	9 (8.4)	10 (19)
Bitter taste	5 (4.7)	2 (3.8)
Bleeding	5 (4.7)#	14 (26)#
Jaundice	5 (4.7)	3 (5.7)
Breathing difficulties	4 (3.7)	6 (11)
Convulsion/seizure	3 (2.8)	4 (7.6)
Facial edema	3 (2.8)	2 (3.8)
Dizziness	2 (1.8)	–
Hearing problem	1 (0.9)	–
Unconsciousness	1 (0.9)	2 (3.8)
Dark urine	1 (0.9)	3 (5.7)
Abortion	1 (0.9)	–
N data points	106	52

Due to a variable number of missing values, the number (n) of data points that were included in the analysis is indicated with each category.

Abbreviations:

a Patients who were discharged after recovery.

b Patients who died during hospitalization.

c 1+, Lassa virus RT-PCR was only positive with undiluted plasma; 2+, Lassa virus RT-PCR was positive with 1/10-volume plasma, irrespective of whether the undiluted sample was positive or not.

# p<0.01 (PCR-positive survived vs. PCR-positive died).

The 1/10-volume sample (defined as 2+ score) was more frequently positive in fatal cases than in survivors (p = 0.001), indicating higher virus load in fatal cases. Clinical symptoms reported at presentation largely matched the known symptoms of Lassa fever [10], [14]–[20]. The only symptom that was significantly more frequent among fatal cases was bleeding (p = 0.0001). Urea and creatinine blood levels were available for a subset of patients. Both values were clearly elevated in fatalities compared to survivors (p<0.0001 for urea and creatinine) (Figure 3).

### Seasonality of Lassa fever

**Figure 4:**
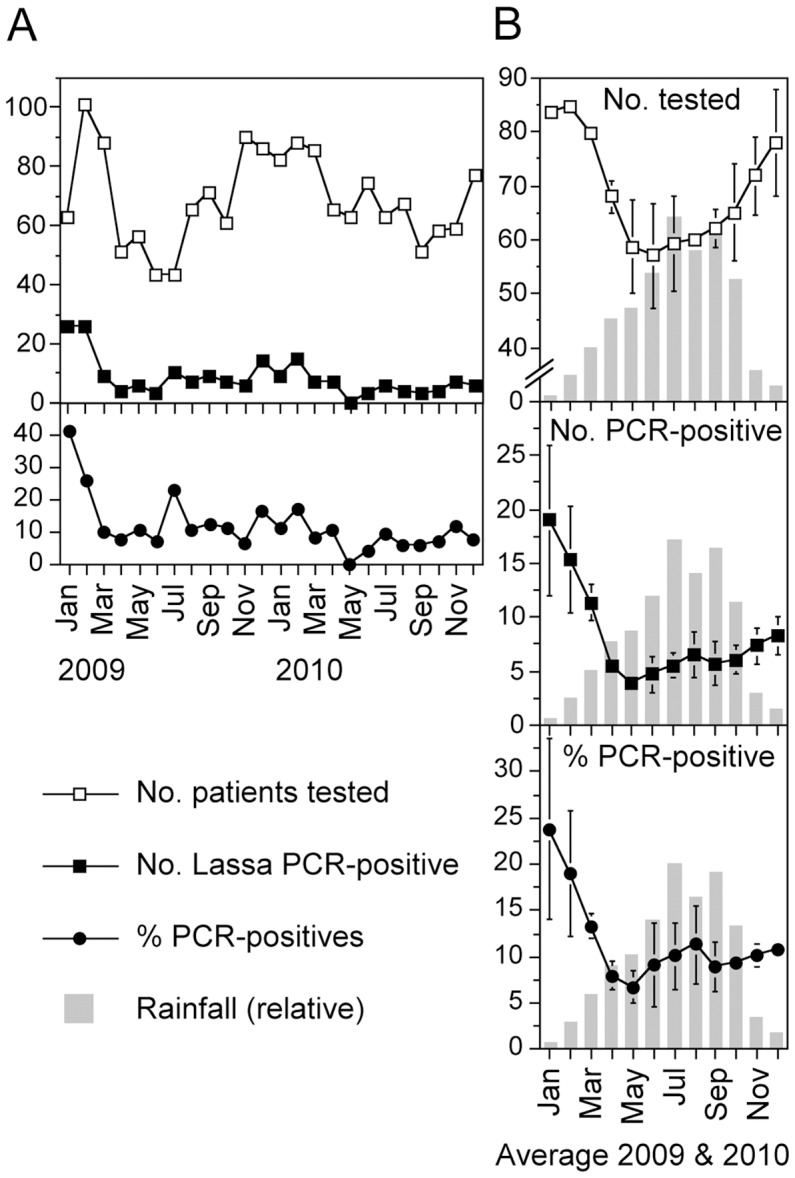
Seasonality of Lassa fever. (A) Number of cases tested, number of positive cases, and percentage of positive cases per month. (B) Average of incidence figures. The curves in A were smoothened by a sliding window covering a 3-month interval and subsequent averaging of the two years. Error bars indicate the range. The rainfall in Benin City is shown as a bar chart in the background in relative units (July = 360 mm). Climate data were taken from http://www.climatedata.eu/climate.php?loc=nizz0004&lang=en (accessed 1 June 2012).

Two levels of seasonality were observed (Figure 4). First, the total number of tests followed a seasonal pattern. From April through October, about 25% fewer patients were tested than in the remaining months. Second, the percentage of Lassa fever positive samples dropped by about 50% in the same period. Both effects led to a seasonal pattern with high Lassa fever incidence in the hospital from November through March (dry season) and low incidence from April through October (rainy season).

### Molecular epidemiology of Lassa virus in Edo State and other parts of Nigeria

**Figure 5:**
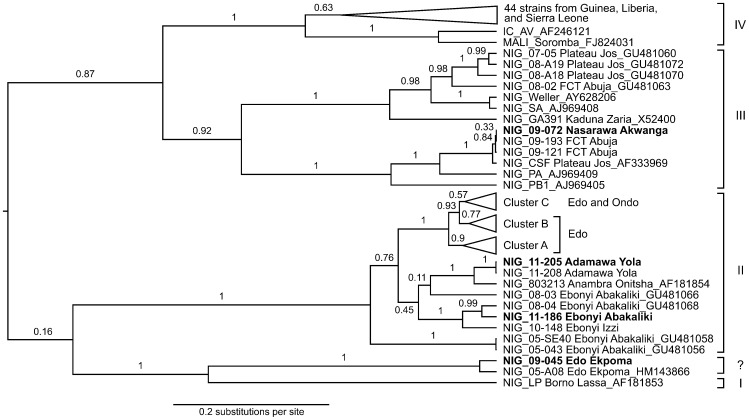
Phylogenetic analysis of Lassa virus sequences. The sequences of the PCR fragments obtained from positive cases were aligned with published sequences. The latter are identified by GenBank accession numbers. For clarity of presentation, only Nigerian strains are shown. The clusters A, B, and C comprising strains from Edo State and Ondo State were collapsed; these strains are shown separately in Figure 6. Posterior probability values are indicated on the branches. The country of origin of Lassa virus strains is indicated by a prefix: IC, Ivory Coast; NIG, Nigeria. If known, State and city is also shown with the strains (FCT, Federal Capital Territory). Sequences highlighted in boldface have been submitted to GenBank (accession nos. JN651366-JN651400). Lassa virus lineages are indicated left. The novel putative lineage/sub-lineage represented by strain Nig05-A08 [49] is indicated with a question mark.

**Figure 6:**
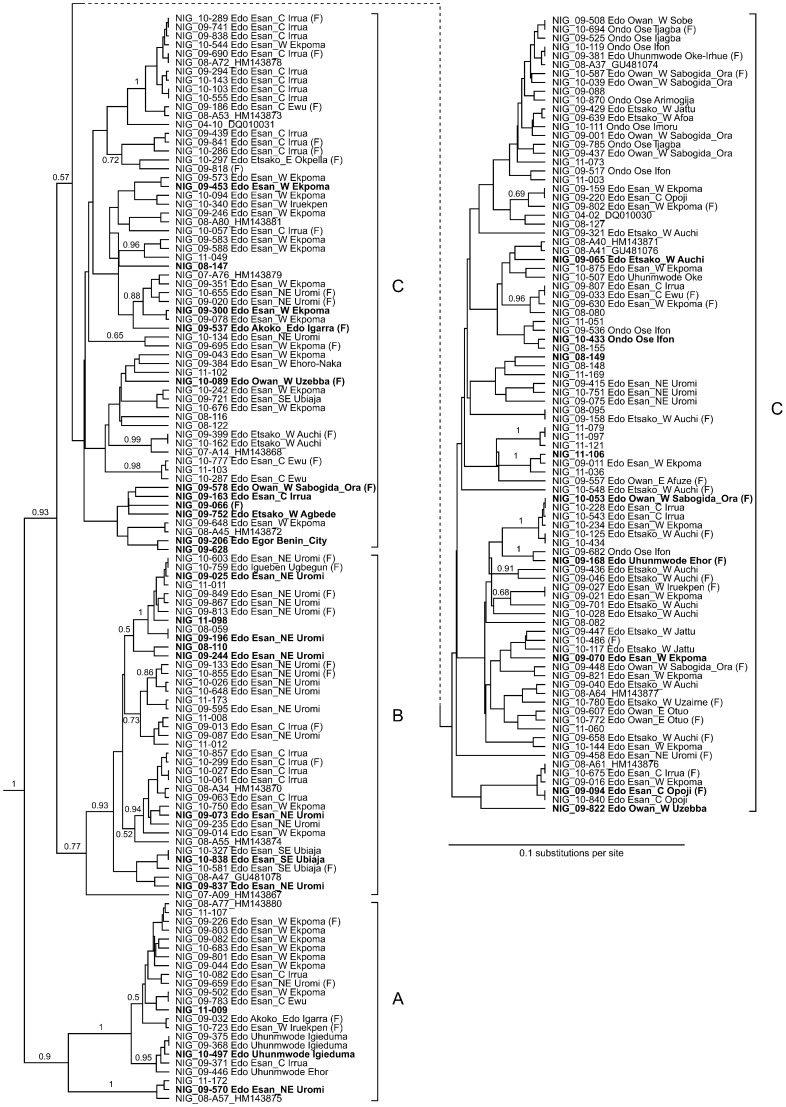
Phylogenetic analysis of Lassa virus sequences from Edo State and Ondo State. Clusters A, B, and C collapsed in Figure 5 are shown in detail here. The upper part of cluster C has been moved to the right (dashed line) to facilitate representation of all sequences. Published sequences are identified by GenBank accession numbers. The posterior probability of monophyly of the corresponding clade is indicated on the branches if the probability is ≥0.5. If known, State, Local Governmental Area (C, Central; W, West; E, East; NE, North-East; SE, South-East), and city is shown with the strains. Sequences from fatal cases are marked with (F). Sequences highlighted in boldface have been submitted to GenBank (accession nos. JN651366-JN651400). Note, the tree contains some negative branch length at nodes with low posterior probability. This is a correct computational result which arises from calculation of the branch lengths in the consensus tree.

The short PCR fragments obtained in routine diagnostics were sequenced and subjected to phylogenetic analysis. Sequences from Edo and Ondo State cluster within lineage II (Figure 5). New sequences obtained from samples sent from Adamawa State (Nig11–205 and Nig11–208 from Yola) and Ebonyi State (Nig11–186 from Abakaliki and Nig10–148 from Izzi) also cluster with lineage II, while sequences of samples from Nasawara State (Nig09–072 from Akwanga) and the Federal Capital Territory (Nig09–121 and Nig09–193 from Abuja) cluster with lineage III. This is in agreement with the known geographical distribution of Lassa virus lineages in Nigeria [3], [49]. In addition, one sequence from Edo State (Nig09–045) was found to cluster with the new putative lineage that was recently described in Edo State and is defined by sequence Nig05-A08 (marked with “?” in Figure 5) [49]. All other sequences from Edo and Ondo State form a separate clade within lineage II that can be further subdivided into three clusters (A, B, and C), although the posterior probability support for cluster C is weak (Figure 6). The analysis of larger sequences might substantiate this tree topology. Strains within these three clusters do not show a strict geographical clustering, presumably because the sequences are too short to further resolve the relationships. However, there are a few visible associations between the origin of the strains and their phylogeny. Cluster A contains strains from Esan West and Uhunmwode, cluster B contains mainly strains from Esan Northeast and Central, while cluster C contains strains from all parts of Edo State as well as the strains from Ondo State. Even though the sequences are too short for an association study, it is worth mentioning that there is no obvious clustering of strains from patients with fatal outcome; they are randomly distributed over the whole phylogenetic tree.

## Discussion

Pilot investigations initiated in 2003 by the University of Lagos, ISTH, and BNI suggested that Edo State is a hot-spot for Lassa fever [31]. These data led to the decision to establish at ISTH a routine diagnostic service for Lassa fever to facilitate appropriate case management. It was further decided to use PCR as it offers case detection at an early stage [24], [25]. The training of staff members of ISTH in theory and practice of diagnostic PCR in partner institutions and on-site has been of paramount importance for the implementation of this technology at ISTH. Featuring separate areas for virus inactivation, RNA extraction, and mastermix preparation, the laboratory complies with basic standards of diagnostic PCR facilities. To guarantee smooth operation of the laboratory, technologies and workflow of the diagnostic facility at BNI in Hamburg were “mirrored” at ISTH, with only minor modification. This strategy facilitated training, interchangeability of protocols, and troubleshooting.

The most recent version of the GPC gene-specific RT-PCR assay [28] was chosen as a PCR assay and samples were tested using the regular extraction volume and 1/10-volume to minimize the problem of PCR inhibition [46]. Indeed, several samples tested positive with the 1/10-volume RNA preparation only and contamination was excluded in most of them by sequencing. It is likely that in these cases amplification of the undiluted sample was inhibited. A modification to the BNI procedures has been the use of diatomaceous earth for RNA preparation instead of a commercial kit to reduce the costs [40].

The main problem that arose during operation of the laboratory was PCR contamination, which was confirmed by sequencing of the PCR products. It was probably facilitated by the high analytical sensitivity of the assay (15 virus genome copies per reaction are detected with a likelihood of 95%) [28] and the need to open the reaction tubes for detection in agarose gel. The staff of the laboratory was aware of this inherent problem of PCR and protocols were developed to minimize and cope with it. We found retrospective evidence for reporting a false positive result in about 1% of the cases tested, which corresponds to an analytical specificity for the whole diagnostic process of 99% and an overall positive predictive value of 90%. However, with one exception, all contaminations manifested in only one of the two reactions (undiluted and 1/10-volume) performed on each sample. Thus, the positive predictive value for the majority of positive samples, namely those positive in both reactions is 99%, while the positive predictive value for samples positive in only one of the two reactions is 80%. Overall, we consider these good performance characteristics for a PCR diagnostics performed in a resource-limited setting.

Complementing the RT-PCR by antibody testing would further improve the reliability of the diagnostics. On the one hand, serology may be used to confirm the PCR diagnosis in patients who have already developed antibodies during the acute phase. On the other hand, antibody testing facilitates detection of patients in the convalescent stage when the virus load has dropped below the detection limit of the PCR [25], [26], [50].

The demographic data of the patients did not provide clues as to risk factors associated with Lassa fever; profession, geographic origin, and gender did not differ significantly between patients who tested positive and those who tested negative. However, a seasonal pattern of Lassa fever incidence was observed with the lowest number of cases during April through October, which corresponds to the rainy season. Similar, though not identical seasonal fluctuations have been described in Sierra Leone and Guinea [14], [15], [51]. The reason for a decrease in incidence during rainy season is not clear. Behavioral changes may play a role, as the number of all patients tested also decreased during this time, which may suggest that patients attend the hospital less frequently in rainy than in dry season. In addition, rodent dynamics and climate factors influencing the efficacy of virus transmission from the reservoir to humans may be involved [2], [52].

The CFR of 31% is high, though in the range of previous reports. In hospitalized patients with endemic Lassa fever, the CFR ranged from 9.3% to 18% [14]–[16], [51], [53], [54]. During nosocomial outbreaks, the CFR appears to be higher, ranging from 36% to 65% [9]–[12]. However, these figures are not fully comparable, due to differences in case definitions and diagnostic methods used in the various studies. Half of the patients with febrile illness attended ISTH at day 5 after onset of symptoms or later, and 50% of those with Lassa fever even at day 6 or later. The efficacy of ribavirin treatment decreases with progression of the disease and is hardly effective after day 6 [23]. Thus, a large number of Lassa fever patients attending ISTH did not benefit as greatly as they could have from the administration of ribavirin early in their disease course. This may explain the CFR of one third. Indeed, the time for therapeutic intervention is extremely short, as 50% of the fatal cases die before day 10 of illness and within 2 days in hospital—often before ribavirin treatment could be commenced. The severity of Lassa fever also explains why most of the patients attend the emergency department rather than the general outpatient departments.

A few parameters were identified which differ significantly between fatal cases of Lassa fever and survivors and are not yet documented in the literature. An important finding was lower body temperature in fatal cases. Often the temperature was not elevated at all or even below the normal range (<35.5°C). This resembles the sepsis-associated hypothermia which is a predictor of poor outcome [55], [56]. Although hypothermia is common in end-stage shock and organ failure of any etiology and not specific for Lassa fever, this sign has implications for the case definition of Lassa “fever”, which apparently needs to be revised to facilitate sensitive detection of cases in the terminal stage. Another factor associated with fatal outcome was higher age. Elderly people are also at higher risk of dying from sepsis, which is thought to be related to a reduced immune status or immune dysfunction [57], [58]. Bleeding was identified as the only clinical sign at presentation associated with poor outcome. Creatinine and blood urea levels were strongly elevated in the fatal cases suggesting renal failure. The semiquantitative PCR data also indicated higher virus load in patients with poor prognosis, which is consistent with published data [26].

The burden of Lassa fever in most regions of Nigeria is not known, as hospitals are not able to detect Lassa fever patients by laboratory testing. In future, surveillance systems including laboratory confirmation in reference centers need to be implemented in the country. Taken together, the data from ISTH indicate that fatal cases of Lassa fever are characterized by the following criteria:

- Presentation within 10 days after onset of symptoms.
- History of fever and/or fever at presentation.
- Attendance of the emergency department.
- Severe systemic disease including renal failure and/or normo- or hypothermia.
- Symptoms include vomiting, diarrhea, abdominal pain, headache, cough, or bleeding.
- Fatal outcome within four days after hospital admission despite antimalaria and antibiotic treatment.

We propose to use the above set of criteria as a surveillance tool to identify hospitals that are attended by Lassa fever patients. While this case definition is less sensitive, as it targets fatal cases only (“the tip of the iceberg”), it may be more specific for Lassa fever than existing ones.

The sequences generated from the short PCR products confirmed previous studies showing that Lassa virus strains from Edo State cluster phylogenetically with lineage II [3], [49]. Although the sequences originate from the same geographical area, they are quite diverse, which is in agreement with previous reports [3], [6]. A first attempt to correlate Lassa virus sequences with outcome did not reveal associations at least on the level of the clades that were resolved by the phylogenetic program. More sophisticated studies are warranted to look into possible links between virus genetics and clinical presentation.

In conclusion, routine diagnostics for Lassa fever has been established at ISTH. There are two major advantages for case management. First, early detection of a Lassa fever case improves protection of staff from nosocomial Lassa virus transmission. In the pre-diagnostic era, an unrecognized Lassa fever patient may have been cared for on a regular ward for several days, before clinical signs raised the suspicion of Lassa fever and appropriate measures were taken. Now, Lassa fever cases are transferred immediately to a specific ward where they are appropriately managed. In addition, close contacts to the Lassa fever patients, including hospital staff, can be monitored or offered ribavirin post-exposure prophylaxis as early as possible if the contact was very close [39]. Second, ribavirin treatment can be commenced early in all Lassa fever cases or may be terminated in non-cases if it had been provisionally commenced on clinical suspicion. Treatment is no longer based on clinical criteria, which is neither sensitive nor specific. However, rapid on-site diagnosis alone probably does not reduce the case fatality rate as long as most Lassa fever patients present too late for ribavirin treatment to be efficacious.

The open PCR platform may be used also for molecular testing for other pathogens. In addition, it provides the basis for research involving the Lassa fever patient and the optimization of the supportive treatment, including renal dialysis and intensive care. Steps to upgrade the laboratory with equipment for viral load determination, serology, blood chemistry, and hematology have been undertaken.
